# Rocker Geometry in Running Footwear: A Scoping Review of Definitions, Commercial Descriptors and Outcome Measures

**DOI:** 10.1002/jfa2.70200

**Published:** 2026-08-19

**Authors:** Priyanka Khusal, Mike Frecklington, Sarah Gardner, Aaron Jackson

**Affiliations:** ^1^ School of Allied Health Faculty of Health & Environmental Sciences Auckland University of Technology Auckland New Zealand; ^2^ Active Living and Rehabilitation: Aotearoa New Zealand Health and Rehabilitation Research Institute School of Clinical Sciences Auckland University of Technology Auckland New Zealand; ^3^ Sports Performance Research Institute New Zealand (SPRINZ) Auckland University of Technology Auckland New Zealand

**Keywords:** athletic equipment innovation, gait, lower limb biomechanics, midsole curvature, rocker, shoes, terminology

## Abstract

**Background:**

Rockered midsoles are increasingly used in running footwear, yet definitions, terminology and outcome measures vary widely across research and commercial sectors.

**Aim:**

This scoping review mapped how rockers are defined in academic literature, how major footwear brands describe them and which outcome measures are most frequently evaluated.

**Methods:**

A scoping review was conducted using systematic searches of four academic databases, identifying 19 eligible studies. Commercial materials from eight leading footwear brands were also examined to assess nonacademic descriptions of rocker features.

**Results:**

Academic sources used diverse terminology and geometric descriptors, such as apex position, rocker angle, curvature radius and toe spring, but no consistent definition or threshold for classifying a rocker was identified. Commercial descriptions relied on qualitative brand‐specific language lacking measurable parameters. Research reported a wide range of outcome measures, most commonly kinematic, kinetic and plantar pressure variables, with fewer studies assessing muscle activation or metabolism and none reporting prospective injury outcomes. This variability reflects differing proposed functions but limits comparability across studies.

**Conclusion:**

Definitions and assessments of rocker designs vary substantially across academic and commercial contexts, limiting the ability to develop evidence‐based clinical recommendations. A standardised measurable definition of rocker geometry would improve comparability between models and enhance clarity when communicating their effects. Future work should prioritise consensus building and examine individual‐level biomechanical and clinical responses to clearly defined rocker designs to better inform clinical recommendations.

## 1 Introduction

Running is a widely adopted form of physical activity, with adult participation rates in Western countries estimated at approximately one in five [1, 2]. The accessibility and relatively low cost make running appealing to many as footwear is often considered the only essential piece of equipment. However, selecting appropriate footwear has become increasingly complex due to the expanding variety of design features, individual preferences and lack of academic or clinical consensus regarding optimal choice [3, 4]. One such footwear design characteristic which is frequently discussed by clinicians is midsole geometry, specifically longitudinal curvature often described as a rocker [5, 6].

**FIGURE 1:**
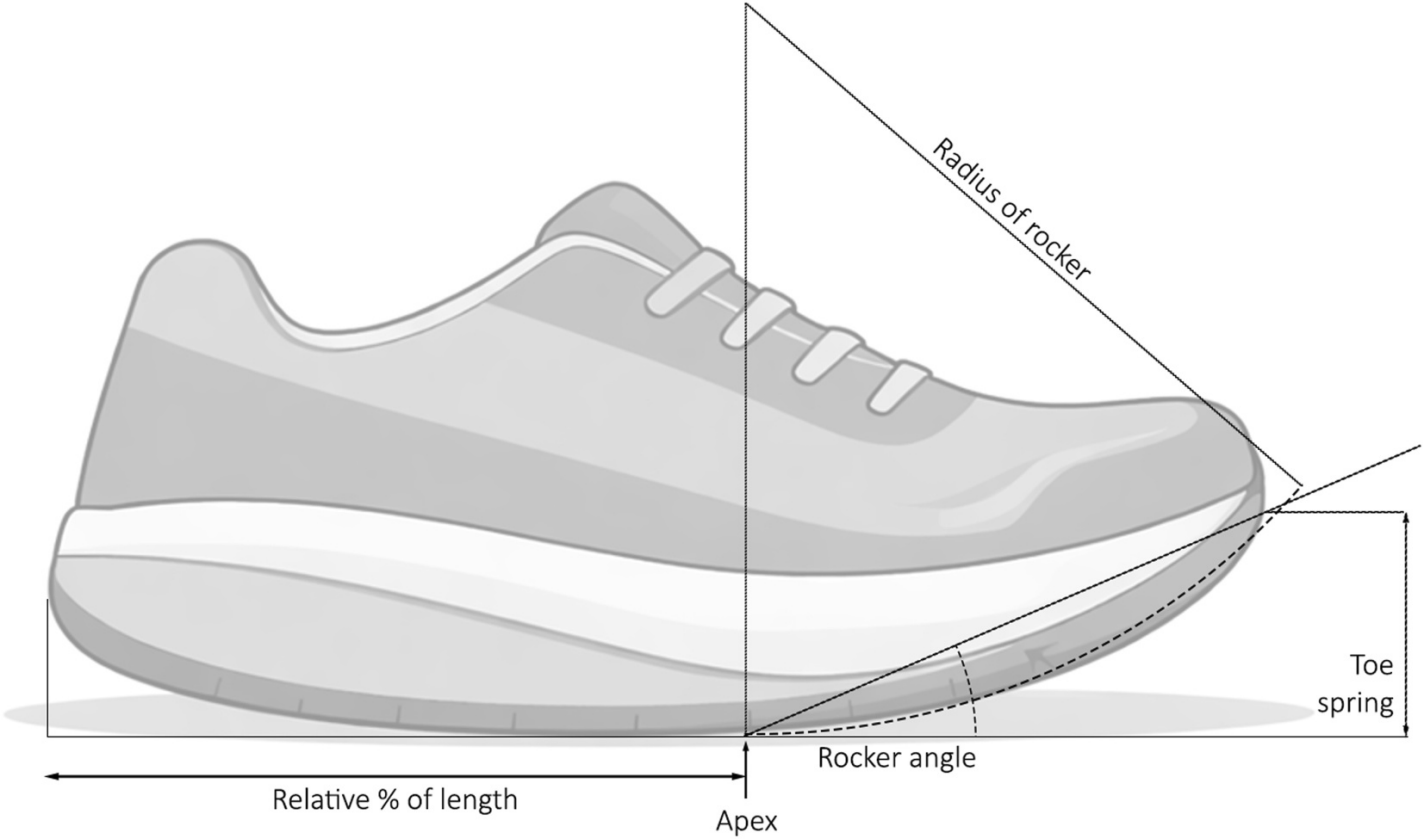
Schematic illustrating geometric parameters used to characterise rocker profiles in running footwear. The apex, defined as the most anterior point maintaining full ground contact under static loading, is expressed as a percentage of total shoe length measured from the posterior reference point. The rocker angle represents the angle between the forefoot curvature and the horizontal ground plane, whereas the rocker radius corresponds to the radius of curvature fitted to the forefoot rocker segment. Toe spring is shown as the vertical elevation of the forefoot above the ground plane, which has been measured variably across studies either from the distal tip of the shoe outsole or from the dorsal‐most point of the toe box.

Rocker soles were originally developed for orthopaedic and therapeutic footwear, where they were used to modify gait mechanics, redistribute plantar pressures and alter joint motion in individuals with musculoskeletal or metabolic conditions, such as diabetes mellitus [7, 8, 9, 10]. Today, clinicians may recommend these geometric features in modern running shoes to actively manage specific mechanical loads. Biomechanically, rocker designs have been purported to alter the sagittal plane function of the foot during stance by substituting physiological joint motion for outsole curvature, with their effects suggested to depend on geometric characteristics (Figure 1.) such as apex position, toe spring, angle and radius of curvature [11, 12].

In running shoes, rockers are commonly incorporated to facilitate forward progression, smooth the transition from midstance to toe‐off and influence the distribution of mechanical work across the lower limb [13, 14]. Experimental studies suggest that rockered running footwear can reduce ankle joint moments and plantarflexor work, potentially decreasing mechanical and energetic demands placed on the Achilles tendon and gastrocnemius‐soleus complex [15, 16, 17]. These biomechanical changes have been associated with context‐dependant improvements in running economy, particularly when rocker designs are combined with compliant midsole materials or increased longitudinal bending stiffness [18, 19]. However, reported effects vary considerably across studies, depending on shoe construction, running speed and participant characteristics [20, 21].

Despite their long‐standing use as a distinct footwear design feature across clinical, experimental and athletic contexts, rocker geometries can be considered independently of other characteristics. Yet in practice, they are typically embedded within complete footwear systems, making it challenging to isolate their specific mechanical and biomechanical effects. Rocker designs are characterised in the literature by substantial inconsistency in terminology and description. Terms, such as rocker sole, rocker geometry, rocker profile, forefoot rocker and toe spring, are often used interchangeably, despite referring to distinct geometric or functional characteristics [8, 13, 22]. This lack of standardisation is evident across the literature, where rocker‐related features are frequently described qualitatively or with insufficient detail to allow replication or meaningful comparison [23]. Furthermore, footwear brands commonly employ proprietary terminology and link rocker features to claims of improved efficiency, propulsion or injury reduction, without clear alignment to research definitions or outcome measures [13, 19].

Given the increasing emphasis placed on rocker designs in running footwear and their promotion as performance‐enhancing, load‐modifying or risk reducing features, a comprehensive synthesis of how rockers are defined, described and evaluated is warranted. To address this need, this scoping review sought to systematically map existing research and commercial material on rockers in running footwear. The aim was twofold, first to identify current definitions and terminology in research‐based descriptions and by popular footwear brands, second, to map the outcome measures most assessed in studies of rockered running shoes, whilst also considering any purported benefits of rocker designs that are highlighted in commercial materials. Three primary research questions guided the development of this study: (1) How are ‘rockers’ defined and described in research on running footwear? (2) How do these definitions compare with descriptions used by popular footwear brands? (3) What outcome measures are most commonly assessed in studies investigating rockered running shoes?

## 2 Methods

The scoping review was undertaken in accordance with the methodological framework outlined by Arksey et al. (2005) [24] and reported following the Preferred Reporting Items for Systematic Reviews and Meta‐Analyses extension for Scoping Reviews (PRISMA‐ScR) guidelines (Supporting Information S1) [25]. Data collection examined two sources (academic literature and commercial data), each with different methods. Firstly, an academic search strategy was collaboratively developed in consultation with a research librarian and systematically applied across four electronic databases: Scopus, MEDLINE via PubMed, SPORTDiscus and CINAHL. The search string used was ((rocker* AND run* AND (footwear OR shoe))), with truncation symbols adapted to each database. No restrictions were imposed on publication date to ensure inclusivity of relevant literature. The search was executed on 9th of December 2025.

Academic sources were included if they used the term ‘rocker’ and involved rockered footwear within the study methodology (as an intervention within trials or as a central component of a review). The search strategy was centred on the term ‘rocker’ to align with the aim of examining how this concept is defined and used in the literature. Broader geometric descriptors (e.g., apex position or curvature radius) were not included as the focus of the review was on terminology and descriptions explicitly referring to rocker designs. This approach prioritised specificity and consistency in study selection. Studies were excluded if they focused on unrelated footwear characteristics, were not written in English or were not available as full‐text articles. Experimental and review studies were included; short communications, conference proceedings and abstracts were excluded. Reference lists of included studies were also screened to identify any additional relevant articles.

Duplicate records were removed using Rayyan (Rayyan Systems Inc., Qatar), followed by manual verification. Two reviewers (PK and AJ) independently screened all titles and abstracts against the inclusion criteria. The same reviewers subsequently assessed the full texts for potentially eligible articles to determine final inclusion. Any disagreements were to be resolved through consultation with a third reviewer; however, this was not required. Data were independently extracted by a single reviewer (PK) and compiled using Microsoft Excel (Microsoft Corp., WA, USA). To ensure accuracy, data were also extracted using Notebook LM (Google, 2024) and contrasted against the manually extracted data.

Commercial data were obtained from ten footwear brands. Nine were selected because they were the most popular among participants in the 2024 Auckland Marathon in New Zealand. These brands were Adidas, ASICS, Brooks, Hoka, Mizuno, New Balance, Nike, On and Saucony. Additionally, Masai barefoot technology (MBT) was included due to it being the only brand of commercially available, unmodified footwear used in academic sources. For each brand, 2025/2026 product catalogues and official websites were reviewed. The lead author (PK) searched these sources during January 2026 for any occurrence of the term ‘rocker,’ and all relevant information was extracted into Microsoft Excel. All brand and product names are used for identification purposes only and remain the property of their respective owners.

## 3 Results

### 3.1 Selection and Characteristics of Sources

**FIGURE 2:**
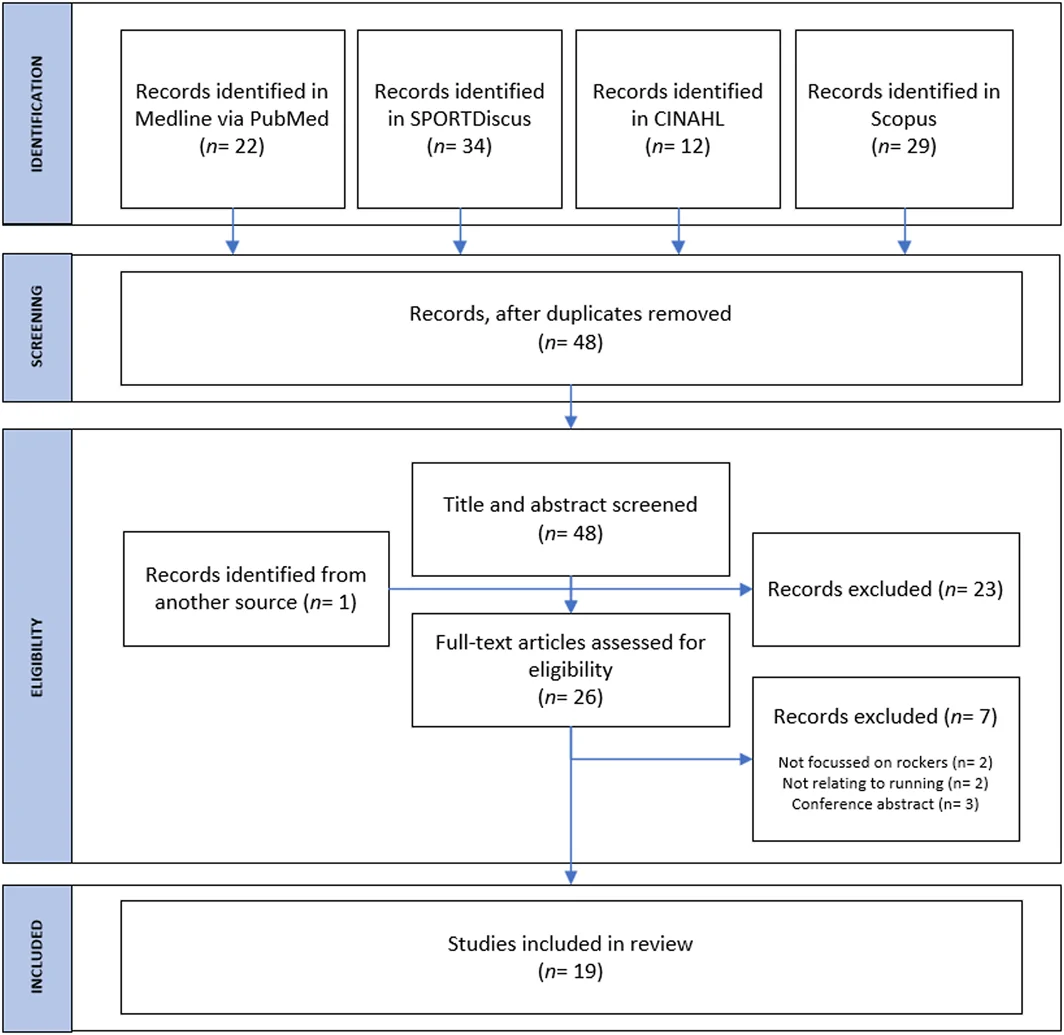
Flowchart of the literature search and screening process.

Figure 2 outlines results of the search and screening process. In addition to studies identified through database searching, one additional study [26] was identified through screening of reference lists of included articles. A total of 48 unique studies were included for screening, with 19 studies meeting the inclusion criteria following full‐text review. All studies were published between 2009 and 2025. Most studies (*n* = 17, 89%) were experimental [15, 16, 26, 27, 28, 29, 30, 31, 32, 33, 34, 35, 36, 37, 38, 39, 40] and only two (11%) were systematic reviews [6, 41]. The experimental studies investigated the influence of rockered footwear in a range of populations including healthy recreational runners [15, 26, 28, 29, 30, 31, 32, 34, 35, 36, 37, 38, 39, 40], those with Achilles tendinopathy [16], a cohort following ankle surgery [27] and endurance runners recovering from exercise induced muscle damage [33]. Although all included studies (*n* = 19) involved rockered footwear, this was applied in different ways across the 17 primary experimental investigations. Eight studies (42%) utilised standard footwear that were extrinsically modified, primarily by adding a stiffened rocker profile [16, 27, 35, 36, 37] or custom components such as carbon plates or orthoses [30, 34]. Five studies (26%) developed custom prototypes specifically for research purposes [26, 32, 38, 39, 40]. Four studies (21%) utilised commercially available options [28, 29, 31, 33], with MBT being the identified brand. The remaining two sources (11%) were systematic reviews [6, 41] that synthesised evidence across these various application methods.

Of the ten commercial footwear brands included in this review, product catalogues for the 2025/2026 season were successfully sourced for eight (80%). Adidas and Nike were the only brands for which researchers were unable to obtain a product catalogue. Public‐facing websites for all nine brands (including Adidas and Nike) were then searched for the appearance of the word ‘rocker.’ At the time of the search, two brands (Mizuno and Nike) did not include the word ‘rocker’ in any catalogue or website reviewed, so are not represented in the results that follow.

### 3.2 Terminology and Language Used to Describe Rockers

Across academic publications, a range of descriptors appeared (Table 1), including rocker shoes [16, 26, 30, 35, 36, 37, 38], rocker soles [34, 41], rocker‐bottom shoes [26, 29, 35, 37, 41], regionalised rockers [40], inverted rockers [34] and unstable rockers [6, 28, 33]. By comparison terminology applied by footwear brands (Table 2) was more heterogeneous, particularly because of proprietary terms with brand specific meaning and application. These included terms such as unique rocker shape (On), curved rubber rocker (Adidas), gradual rocker sensation (Saucony), GlideRoll rocker (Brooks), meta‐rocker and speed centric meta‐rocker (Hoka) and agile forefoot rocker (On).

**TABLE 1:** Key details extracted from academic sources.

Author (year)	Study design	Population	Terminology applied	Definition	Commercial/Added rocker	Design features/Measurement	Visual reference/picture	Investigated function
Arazpour et al. (2025) [41]	Systematic review	Adult human participants (≥ 18 years), both asymptomatic and with lower‐limb pathologies	Rocker sole shoes; rocker sole and rocker‐bottom shoes	Rocker sole footwear is defined as shoes incorporating a curved sole profile designed to alter foot biomechanics by off‐loading stressed anatomical structures, redistributing plantar pressure, modifying foot and ankle kinematics and facilitating a smoother gait cycle. Rocker soles are classified into distinct types (e.g., toe‐only, heel‐to‐toe, double, and negative heel) based on the position of the rocker apex, with each configuration intended to elicit specific biomechanical effects	Commercially available and custom‐made rocker soles	Includes multiple rocker configurations: Toe‐only, heel‐to‐toe, heel and forefoot, double rocker and negative rocker designs. Key design parameters include rocker apex position (typically 50%–65% of shoe length), rocker angle/radius, longitudinal bending stiffness and sole material properties. Outcomes synthesised across walking and running tasks using kinematics, kinetics, plantar pressure, EMG and spatiotemporal measures	Yes	Effects of rocker sole shoes on lower‐limb biomechanics during walking and running, including joint kinematics, joint moments and power, muscle activation, plantar pressure redistribution and spatiotemporal gait parameters to inform clinical prescription and customisation
Boyer et al. (2009) [28]	Cross‐over trial	*N* = 19 healthy adults (57.9% female); mean age: women 28.9 ± 7.3 yrs and men 32.6 ± 7.5 yrs	Rockered shoe; in‐sole rocker; sole rocker and anterior–posterior rocker	A rockered shoe defined as footwear incorporating a rounded sole profile in the anterior–posterior direction, creating instability that alters ankle joint mechanics during running	Commercial rocker shoe	Masai barefoot technology (MBT) M‐walk shoe (625 g), characterised by a rounded anterior–posterior sole profile creating AP instability. Sole composed of two materials: one under the heel and a different material under the anterior sole. Compared against a standard running shoe (new Balance 658; 269 g)	Yes	Mechanisms of adaptation to rockered sole shoes during running, specifically changes in lower‐limb kinematics and kinetics at the ankle, knee and hip joints, including joint angles, ground reaction forces, joint moments, joint power, stance time and running speed
Chen et al. (2022) [29]	Cross‐over trial	*N* = 17 healthy young males (age 22.0 ± 1.6 yrs)	Rocker‐soled shoes; rocker shape and rocker profile	Rocker‐soled shoes are defined as footwear incorporating a curved sole profile, including a rounded heel and toe rocker, functionally designed to influence lower‐limb kinematics and muscle activation during walking, running and functional movements	Commercial rocker shoe	One rocker‐soled shoe and one normal‐soled shoe of the same brand were tested. The rocker profile was similar to MBT‐style shoes, incorporating a rounded heel and toe rocker. Shoe mass was closely matched to minimise weight effects (rocker: 293.8 g and normal: 262.0 g). Both shoe types had similar sole material properties, stiffness and hardness (shoe size US 9 and length 27 cm)	Yes	Effects of rocker‐soled shoes on lower‐extremity biomechanics during functional tasks, including hip, knee and ankle joint kinematics across sagittal, frontal and transverse planes and muscle activation patterns (vastus medialis, vastus lateralis, biceps femoris and gastrocnemius) during walking, running, stair negotiation, cutting and jumping
Frigg et al. (2016) [27]	Cross‐over trial	Adults with total ankle replacement (TAR), ankle arthrodesis (AA), tibiotalocalcaneal arthrodesis (TTC) and healthy controls	Rocker‐bottom shoes	Rocker‐bottom shoes are defined as footwear incorporating a stiff curved rocker element attached to the sole to alter foot rollover mechanics and redistribute plantar loading during walking	Added rocker	A standardised new Balance 926 orthopaedic running shoe was used and converted into a rocker‐bottom shoe by attaching a rocker‐shaped stiff plastic element to the sole using Velcro. Comparisons were made between barefoot, standard running shoes and rocker‐bottom shoe conditions	Yes	Effects of rocker‐bottom shoes on walking biomechanics across clinical and healthy populations, assessed using the relative midfoot ndex (RMI), forefoot maximal force, and walking speed to evaluate rollover function, forefoot loading and gait performance following ankle and hindfoot surgery
Freitag et al. (2023) [30]	Cross‐over trial	*N* = 15 healthy recreational male runners (age 38.5 ± 10.9 yrs and BMI 23.7 ± 1.8); free of lower‐extremity injury or disease	Rocker shoe; curved rocker shoe and heel and forefoot rocker	Rocker shoes are defined as footwear with a curved sole profile, particularly at the forefoot, designed to modify ankle joint mechanics, reduce plantarflexion demands and influence muscle activation during running	Commercial rocker shoe with customised modifications	Commercial Scott Palani heel and forefoot rocker running shoe used as a base model. Three shoe conditions (A, B, and C) differed only in forefoot curvature, achieved by inserting custom‐made carbon plates with varying radii into the forefoot region. Shoe A reflected the original curvature and Shoes B and C incorporated progressively altered forefoot curvature	Yes	Effects of heel and forefoot rocker shoe curvature on pelvic abductor muscle activity (glutaeus medius and glutaeus maximus) during running under different conditions (flat laboratory surface, treadmill running at 0%, 24% uphill and 24% downhill gradients)
Han et al. (2025) [31]	Cross‐over trial	*N* = 15 healthy adults (66.7% male); males age 24.2 ± 2.2 yrs and females age 22.4–23.4 ± 1.2–1.3 yrs	Rocker‐bottom shoes (RBS)	Rocker‐bottom shoes are defined as footwear with thick sagittally curved soles that induce a rolling motion to substitute for ankle joint movement, thereby reducing ankle motion demands and altering hindfoot mechanics during walking	Commercial rocker shoe	Rocker‐bottom shoes characterised by thick sagittally curved soles. Footwear conditions included barefoot, running shoes, high heels, rocker‐bottom shoes and climbing shoes. High‐precision biplanar fluoroscopy was used to quantify talocrural and subtalar joint kinematics during walking	Yes	Effects of rocker‐bottom shoes on ankle and hindfoot biomechanics during walking, including talocrural and subtalar joint dorsiflexion–plantarflexion range of motion, peak joint angles and dynamic hindfoot posture measured using biplanar fluoroscopy
Li et al. (2024) [32]	Computational modelling study (finite element simulation)	Single healthy adult (male, 24 yrs; 60 kg and 175 cm)	Toe spring; rocker shoes	Toe spring is defined as the curvature of the shoe toe and the shoe last, characterised by the upward curve at the forefoot of the sole and the height of the shoe toe above the ground in the finished shoe	Added rocker	Custom running shoes (size 41) with EVA midsole and rubber outsole. Two toe spring conditions were tested: low toe spring (6.5 cm) and high toe spring (8.0 cm) Finite element simulations evaluated metatarsal and hallux stress under different forefoot landing angles during running	Yes	Effects of toe spring magnitude and forefoot landing angle on metatarsal and hallux stress distribution during running, with a focus on identifying configurations that minimise injury‐related stress in the forefoot
Lin et al. (2017) [26]	Cross‐over trial	*N* = 11 healthy adults (0% female; age 32.8 ± 3.1 years; body mass 72.1 ± 6.9 kg; height 173 ± 5.3 cm and shoe size 20.8 ± 2)	‘Rocker‐soled shoes’, ‘rocker designs’, ‘rocker shoe‐soles’, ‘curved shoe‐soles’ and dual rocker	Customised foot orthoses with a specially curved outer sole designed to allow smooth progression through the stance phase, reduce overbending of foot joints and reduce local impacts and stresses	Added rocker	Dual rocker design with frontal rocker angle 20° and rear rocker angle 30°; apex position at 60% (front) and 25% (rear) of shoe length from heel and apex angle 95°. Three‐layer sole construction: Upper midsole EVA I (0.77 MPa) and bottom midsole EVA II (0.54 MPa for Rocker‐1 and 0.77 MPa for Rocker‐2), rubber outsole (4.46 MPa). Compared against barefoot and flat‐soled shoes	Yes	Plantar force (% body weight), difference in plantar force (DPF), temporal gait events (T1, T2), stance phase duration (DSP), overall duration (OD), ankle dorsiflexion angle, foot‐to‐floor angle and therapeutic and preventative function to reduce plantar loading and stress concentration during walking and jogging
Mei et al. (2019) [40]	Cross‐over trial	*N* ≈ 25 habitually shod, rearfoot‐striking runners (healthy young males; age 23.6 ± 2.1 yrs; height 173 ± 4.6 cm and body mass 68 ± 5.8 kg)	Rocker structure; regionalised rocker; unstable rocker structure; rocker stiffness and unstable rocker shoes	An unstable hemispherical rocker element fixed to the outsole beneath the hallux region, intended to modify forefoot mechanics and stimulate toe‐gripping function by altering the application point of ground reaction forces during running	Added rocker	Hemisphere rocker: 4 cm diameter, 1 cm height, positioned at the hallux region. Two stiffness conditions: Hard (*E* = 3.36 MPa and hardness ≈ 67.9 HA) and Soft (*E* = 0.37 MPa and hardness ≈ 8.44 HA). Control shoe: flat, soft sole, no heel‐toe drop or toe spring. Measurements: Lower‐limb kinematics, plantar pressure (force, peak pressure and force–time integral), stance time and running speed	Yes	Gait kinematics, plantar pressure distribution, stance time, running speed and forefoot/toe loading, with emphasis on stimulating toe‐gripping function and altering forefoot biomechanics during running
Munim et al. (2025) [6]	Systematic review	Healthy adult participants (≥ 18 years)	Rocker‐bottom shoes; outsole rocker; forefoot‐only rocker; rearfoot‐only rocker and rearfoot‐to‐forefoot rocker	Footwear with a curved outsole profile, typically thickened midsole and rounded forefoot/rearfoot, designed to facilitate smoother roll‐off during gait; categorised by apex position	Commercially available shoes including MBT, skechers and others	Rocker type: forefoot‐only, rearfoot‐only, rearfoot‐to‐forefoot; toe apex position (TAP) and Heel apex position (HAP) as % of shoe length; toe spring (TS) and heel spring (HS) as mm; toe rocker angle (TRA) and Heel rocker angle (HRA) in degrees and assessed gait kinematics/kinetics, GRF, plantar pressure, muscle activity, spatiotemporal parameters, oxygen consumption and comfort	Yes	Effects on lower‐limb biomechanics during walking and running: Joint angles, moments and power; plantar pressure; muscle activity; spatiotemporal parameters; oxygen consumption and perceived comfort
Nakagawa et al. (2018) [33]	Cohort trial	∼25 healthy adults (40% female)	Unstable rocker shoes; MBT shoes and rocker shoes	Unstable rocker shoes produced by Masai barefoot technology (MBT) are characterised by a rounded sole and soft cushioned heel. The underlying concept is that induced instability increases muscle activity, which is proposed to train functional movement systems, strengthen locomotory muscles and improve balance	Commercial unstable rocker shoes (Masai Barefoot technology, Switzerland)	Rounded sole with soft cushioned heel designed to create instability during standing and walking compared against ordinary shoes. Outcomes included muscle hardness, maximal voluntary isometric joint torque of lower limb muscles and subjective fatigue during recovery from marathon‐induced muscle damage	Yes	To investigate whether wearing unstable rocker (MBT) shoes enhances recovery from marathon‐induced muscle damage by accelerating restoration of muscle hardness, strength and perceived fatigue
Sánchez‐Gómez et al. (2020) [34]	Cross‐over trial	21 healthy adults (52.4% female)	Inverted rocker; inverted rocker orthoses; rocker‐sole footwear and rocker‐bottom soles	Inverted rocker orthoses are described as EVA rocker elements placed directly beneath the first metatarsophalangeal joint (IMTPJ), intended to restrict IMTPJ motion and alter the windlass mechanism, thereby influencing triceps surae muscle activity during running	Added rocker	NIRO: EVA inverted rocker element (5 cm long × 2 cm wide × 6 mm or 8 mm thick), smoothly polished edges, positioned under the IMTPJ. TMEO: Total motion‐restricting orthosis with similar thicknesses (6 and 8 mm), semi‐rigid EVA. Five conditions compared: Shoe‐only (SO), NIRO‐6 mm, NIRO‐8 mm, TMEO‐6 mm and TMEO‐8 mm	Yes	To compare the effects of NIRO versus TMEO inverted rocker orthoses on electromyographic (EMG) activity of the gastrocnemius medialis and lateralis during running
Sobhani et al. (2013) [36]	Cross‐over trial	*N* = 16 (50% female; age 29 ± 9 years; height 177.1 ± 9.3 cm and body mass 69.8 ± 11 kg); healthy adults	Rocker shoe; rocker profile; stiffened rocker profile; rocker radius; toe rocker and stiffened rocker sole	‘Biomechanically, rocker shoes with the apex proximal to the metatarsophalangeal joint cause a decrease in the external dorsiflexion moment arm of the ground reaction force around the ankle joint. This alteration reduces the external dorsiflexion moment and consequently results in smaller plantarflexion moments around the ankle.’	Added rocker	Apex (rolling point) of rocker shoes positioned at 53% of shoe length (proximal to metatarsal region) compared to 65% in baseline shoes. Rocker profile thickness 2.2 ± 0.1 cm at the apex and under the heel. Shoe mass: baseline shoes 467 ± 87 g and rocker shoes 805 ± 157 g. Custom‐made shoes modified with a stiffened rocker profile	Yes	Ankle joint biomechanics during slow running and walking: primary outcome was peak ankle plantarflexion moment during terminal stance. Secondary outcomes included ankle plantarflexion impulse, ankle power, ankle angles, knee and hip joint moments and EMG (timing and amplitude) of triceps surae and tibialis anterior
Sobhani et al. (2014) [37] (Effect of rocker shoes on plantar pressure pattern in healthy female runners)	Cross‐over trial	*N* = 18 (100% female; age 23.6 ± 3 years; height 171.5 ± 6 cm and body mass 61.7 ± 7 kg); healthy endurance runners	Rocker shoes; rocker profile shoes; rocker‐bottom and stiffened rocker profile	‘Stiffened rocker profile shoes with the apex positioned proximal to the metatarsal heads, intended to alter the rollover process during running and reduce forefoot loading.’	Added rocker	Apex (rolling point) positioned at 53% of shoe length (proximal to metatarsal region) compared with 65% in standard running shoes. Rocker profile thickness 2.2 ± 0.1 cm at the apex and under the heel. Shoe mass: standard running shoes 541 ± 44 g and rocker shoes 858 ± 96 g. Custom‐modified shoes with a stiffened rocker profile	Yes	Plantar pressure distribution during running, including peak pressure (PP), maximum mean pressure (MMP) and force–time integral (FTI) across foot regions, as well as perceived shoe comfort and exploratory analysis of strike pattern effects
Sobhani et al. (2014) [37] (Rocker shoe, minimalist shoe and standard running shoe: A comparison of running economy)	Cross‐over trial	*N* = 18 (100% female; age 23.6 ± 3 years; height 171.5 ± 6 cm and body mass 61.7 ± 7 kg); healthy endurance runners	Rocker shoes; rocker‐bottom shoes; rocker profile and rocker sole	‘Rocker shoes were modified from standard shoes with a stiffened rocker sole by a certified orthopaedic shoe technician. The apex (rolling‐point) positioned proximal to the metatarsal region to reduce plantar flexion moment and forefoot loading during running.’	Added rocker	Apex at 53% of shoe length versus 65% in standard running shoes. Rocker profile thickness 2.2 ± 0.1 cm at apex and under heel. Shoe mass: rocker shoes 858 ± 96 g; standard shoes 541 ± 44 g and minimalist shoes 321 ± 25 g	Yes	Metabolic and physiological responses during running: VO_2_, VCO_2_, respiratory exchange ratio (RER), heart rate (HR) and rate of perceived exertion (RPE)
Sobhani et al. (2015) [16]	Cross‐over trial	*N* = 13 (85% female; age 48 ± 14.5 years; height 172 ± 7 cm and bodyweight 77 ± 14 kg); all participants with Achilles tendinopathy	Rocker shoe; rocker sole and rocker bar	‘During the roll‐off, the application point of the ground reaction force is normally located at the metatarsophalangeal joint with standard shoes. With rocker shoes, however, the application point is applied at the rocker apex instead, proximal to the MPJ.’	Added rocker	Apex (rolling point) of standard shoes at 65% of shoe length and rocker shoes at 53% (proximal to metatarsal region). Rocker profile thickness 2.2 ± 0.1 cm at the apex and under the heel. Shoe mass: standard shoes 467 ± 87 g and rocker shoes 805 ± 157 g	Yes	Gait kinematics, kinetics and spatiotemporal outcomes (ankle plantarflexion moment, knee flexion moment, hip flexion moment, ankle power and ankle dorsiflexion), EMG (gastrocnemius, soleus and tibialis anterior), speed, step length, cadence, stance time and pain
Sobhani et al. (2017) [15]	Cross‐over trial	16 experienced female endurance runners	Rocker shoes; rocker sole and rocker‐bottom	Rocker shoes were standard running shoes modified with a stiffened rocker profile, characterised by a proximally located apex (rolling point) intended to alter lower‐limb biomechanics during running	Added rocker	Apex location: 53% of shoe length (proximal to metatarsal region) versus 65% in standard shoes. Rocker thickness: 2.2 ± 0.1 cm at apex and heel. Shoe mass (pair): Rocker 858 ± 96 g and standard 541 ± 44 g. Primary outcomes: Joint work (positive, negative and net; J/kg) at ankle, knee and hip; joint moments (Nm/kg and Nm·s/kg); kinematics (°) and stance time	Yes	Lower‐limb biomechanics during running, including joint work distribution (ankle, knee and hip), joint moments, kinematics, stance time and influence of strike pattern (rearfoot vs. midfoot) on rocker shoe effects
Tankink et al. (2024) [38]	Cross‐over trial	*N* = 10 (20% female; age 22 ± 1.8 years; height 183 ± 8.5 cm and weight 77.7 ± 11.1 kg); recreational runners	Rocker shoes; rocker profile; apex position and apex angle	‘Experimental rocker shoes with adjustable apex position and apex angle to maximise positive ankle work and redistribute joint work from hip and knee to ankle to improve running economy.’	Added rocker	Outer sole removed, 3 carbon fibre layers with rails/sliders to adjust apex (40%–90% shoe length). Polyethylene layer of 3 mm for protection. Shoe mass: experimental 407–560 g; control 197–261 g. Carbon stiffness: 83.6 N/rad versus 1.9 N/rad in control. Apex angle adjustable	Yes	Running biomechanics: positive/negative ankle work, share of joint work (%), ankle angles, angular velocity, plantar flexion moments, power generation, vertical and anterior‐posterior GRF, metabolic power, step frequency, stance time, running economy
Trama et al. (2019) [39]	Cross‐over trial	*N* = 20 (0% female; age 23.9 ± 2.1 years; height 177 ± 0.05 cm and weight 73.6 ± 7.4 kg); healthy recreational rear‐foot strikers	Rocker shoes and nonrocker shoes	‘Rocker shoes specially designed with greater rear and front elevation of the sole and smaller ground contact surface compared to non‐rocker shoes’	Added rocker	Shoe characteristics for US size 9 (270 mm): Nonrocker versus rocker shoe; mass 237 versus 241 g; EVA sole 40–45 C; mean thickness 48 versus 50 mm; front apex 21% versus 27%; rear apex 14% versus 20% and ground contact 65% versus 53%	Yes	Running biomechanics: EMG of gastrocnemius and vastus lateralis (preactivation and stance), ground reaction forces (impact peak, active peak, VLR and impact frequency), soft tissue vibrations (Accpeak, Pmean, Pmax, HFratio and DAMP) and effect of running speed

**TABLE 2:** Key details extracted from commercial sources.

Brand	Terminology associated with ‘rockers’	Functional descriptions	Measurements and definitions	Associated models
Adidas	Rocker; rocker shape; rocker geometry; forefoot rocker; curved rocker shape; rocker point; forefoot rocker geometry; curved rubber rocker and dynamic rocker design	Enables smooth and efficient forefoot running (especially on trails); promotes smooth roll‐off for quicker pace and extended comfort; ‘forefoot rocker kicks in to rocket you forward’; rocker triggers forward momentum and pushes you onto the next step; enhances forward propulsion; improves running economy; smooths transitions from heel strike to toe‐off; supports speed and performance and keeps you moving comfortably all day	Rocker described qualitatively in terms of propulsion, smooth transitions, forward momentum, comfort and efficiency. No explicit geometric definitions or quantified rocker measurements (e.g., apex location, angle and curvature) provided	Terrex Agravic Speed Ultra Trail Running Shoes; Adistar; Adizero Aruku Shoes and Adizero Adios Pro 4 Shoes
ASICS	Rocker and rockered	Balanced rocker helps propel the runner through the stride; provides a smooth efficient ride; creates propulsion to move the stride forward; supports speed development and suitable for daily mileage, long runs, workouts and some races	Rocker described qualitatively and no explicit rocker measurements or geometric definitions provided	GLIDERIDE 3; EVORIDE SPEED; TRABUCO MAX 2; NOVABLAST 3; SUPERBLAST; EVORIDE and MEGABLAST
HOKA	Rocker; rocker profile; forefoot rocker; forefoot rocker profile; meta‐rocker; smooth meta‐rocker; speed‐centric meta‐rocker and rocker integrity technology	Encourages effortless strides; facilitates smooth transitions through the gait cycle; supports a natural gait; creates smooth heel‐to‐toe roll; enables aggressive toe‐off; pace‐pushing propulsion; supports racing performance; maintains control during long‐haul efforts and promotes confident movement on uneven terrain	Rocker concepts described qualitatively; references to adjustable or curved meta‐rocker geometry; mentions of forefoot emphasis and toe‐off behaviour and no explicit numerical rocker measurements or geometric definitions provided	Mafate (Men's and Women's); Mafate 5; Mafate Speed 2; Skyflow (multiple variants); Mach X 2; Mach X 3; Cielo X1/Cielo X1 2.0/Cielo X 3 LD; Arahi 8; Challenger 8; Clifton 8; Clifton 10 and Bondi 9
MBT	Rocker; rocker sole; mid‐rocker; rocker technology; rocker sole technology; pivot axis; dual‐sensor rocker and Levels 1–3 rocker	Enables smoother roll‐off during walking and running; promotes muscle activation; enhances gait efficiency and posture; reduces joint stress; improves cushioning and comfort; supports all‐day wear and suitable for various activities including walking, running and casual use	Rocker geometry described qualitatively; Levels 1–3 rocker for low, medium or high pivot; mid‐rocker or pivot strike areas referenced; patented rocker sole with sensor technology and pivot axis and no explicit numerical measurements provided	MBT MBT‐2000 II Lace Up and MBT MTR‐1500 II Lace Up
New Balance	Rocker profile	Provides smoother natural‐feeling transitions from heel‐to‐toe	Rocker profile referenced qualitatively and no explicit rocker geometry, angles or numerical measurements provided	Fresh Foam X 860v14 (multiple colourways); Fresh Foam X 1080v14 (multiple colourways); FF More v6 and TCS NYC Marathon FuelCell SuperComp Elite v5
On	Unique rocker shape; agile forefoot rocker; rocker shape; bold rocker shape; pronounced rocker motion; rocker‐shaped and rocker	Promotes effortless forward momentum; provides propulsive toe‐off; delivers a fast propulsive ride; creates a strong heel‐to‐toe roll; supports smoother heel‐to‐toe transitions; enables powerful take‐off and enhances propulsion when combined with carbon Speedboard	Rocker described qualitatively; emphasis on forefoot toe spring, heel‐to‐toe roll and propulsion and no explicit numerical rocker measurements or geometric definitions provided	Cloudmonster; Cloudsurfer; Cloudsurfer Next; Cloudflow 5; Cloudvista 2 and Cloudboom Echo 3
Saucony	Gradual rocker sensation	—	Rocker described qualitatively and no explicit rocker measurements or geometric definitions provided	Kinvara Pro Wide

### 3.3 Definitions of a Rocker

Across the academic literature, rocker designs were described using explicit geometric measures that characterise how the shoe outsole differs from a flat profile (Table 1). The most consistently reported parameter was apex position, typically expressed as a percentage of shoe length. Multiple studies modified footwear to position the apex at 53% of shoe length, proximal to the metatarsal region [16, 36, 37]. Trama et al. (2019) reported both forefoot and rearfoot apex locations at 27% (relative to the front of the shoe) and 20% of shoe length (measured from the back of the shoe), whereas Tankink et al. (2024) developed an adjustable rocker system with an apex ranging from 40% to 90% of the shoe length.

Other geometric parameters used to define rockers include rocker angles and curvature radii. Lin et al. (2017) described a dual rocker design with 20° forefoot and 30° heel rocker angles, whereas Freitag et al. (2023) altered forefoot curvature by inserting carbon plates with progressively different radii (toe spring ranging from 33 to 60 mm). Several studies specify rocker profile thickness, for example, studies by Sobhani et al. (2015), Sobhani et al. (2014) and Sobhani et al. (2014) used identical designs and reported a rocker profile thickness of 2.2 ± 0.1 cm from the heel to the apex. This measurement considered only the added EVA, not the outsole or existing midsole of the modified shoes. Distinct rocker configurations are also defined according to apex placement and curvature distribution, including toe only, heel to toe, double, negative heel and inverted rockers [34, 41]. Additional definitions, such as toe spring height, have been operationalised using direct numerical measurements, as in Li et al. (2024), who compared 6.5 and 8.0 cm toe spring (measured from the dorsal apex of the toe, to the ground) conditions in a custom running shoe. Across studies, certain geometric parameters, most notably apex position, and rocker angle or curvature radius, were more well reported, although measurement approaches and definitions remained variable. In contrast, no studies defined clear thresholds or criteria for classifying footwear as incorporating a ‘rocker’, and reporting of such classification criteria was absent across the literature.

Collectively, academic definitions emphasise quantifiable geometric features, including apex location, rocker angle, curvature radius, profile thickness and stiffness, as the basis for defining rocker designs. However, the absence of an agreed framework for how these parameters should be combined or interpreted limits their application in consistently defining rocker footwear.

In contrast, commercial sources describe rockers qualitatively, without providing geometric measurements (Table 2). Brands frequently reference general concepts, such as a ‘rocker shape’, ‘rocker profile’ or ‘curved rocker geometry’, often paired with performance‐oriented claims. Adidas, for example, refers to ‘forefoot rocker geometry’, a ‘curved rubber rocker’ and a ‘Dynamic Rocker design’ intended to ‘trigger forward momentum.’ Brooks describes its ‘GlideRoll Rocker’ as promoting ‘smooth heel to toe transitions,’ whereas HOKA frequently uses the term ‘MetaRocker’ to denote smoother roll off or enhanced propulsion. On Running describes ‘agile forefoot rocker’ structures that provide a ‘propulsive ride,’ and Saucony refers more generally to a ‘gradual rocker sensation.’ Although some brands mention structural features, such as toe spring or heel bevelling, these are not linked to explicit rocker parameters such as apex position, rocker angle or curvature radius.

### 3.4 Outcome Measures Investigated in Studies of Rockered Running Shoes

Across academic studies, outcome measures used to evaluate rockered footwear span kinematic, kinetic, plantar pressure, muscle activation, spatiotemporal and metabolic domains. Most studies assessed several domains concurrently. Key findings of the included studies are not synthesised in this review but can be found in Supporting Information S2.

Kinematic and kinetic measures were the most frequently reported outcomes, presented by 10 (53%) [6, 26, 28, 29, 35, 36, 37, 39, 40, 41] and 11 (58%) [6, 26, 27, 28, 33, 35, 36, 37, 39, 40, 41] studies respectively. Six studies (32%) assessed ankle, knee and hip joint mechanics, including joint angles, joint moments, joint power and angular velocities [6, 15, 16, 28, 38, 41]. Specific variables reported include peak ankle plantarflexion moment, plantarflexion impulse, ankle dorsiflexion angle and hindfoot motion. Plantar pressure outcomes were commonly assessed, including peak pressure [6, 37, 40, 41], maximum mean pressure [37, 41], force–time integral [37, 40, 41], regional load distribution [6, 40, 41] and forefoot maximal force [27, 40]. Four studies [15, 16, 36, 38] evaluated changes in the location of the ground reaction force application, particularly in relation to the rocker apex.

Muscle activation was examined using surface EMG for muscles such as the gastrocnemius, soleus, tibialis anterior, vastus medialis, vastus lateralis, biceps femoris, glutaeus medius and glutaeus maximus [29, 30, 34]. Some studies specified activation timing (e.g., preactivation, stance‐activation and stance‐phase activation) [16, 30, 36, 39, 41], whereas others focused on EMG amplitude [16, 29, 30, 34, 36].

Spatiotemporal measures were reported by nine studies (47%) [15, 26, 27, 28, 31, 33, 36, 38, 40], including running speed, step length, cadence, stance duration, double‐support duration and temporal gait events.

A smaller number of studies (*n* = 4, 21%) included metabolic or physiological outcomes, such as submaximal oxygen consumption (VO_2_), carbon dioxide production (VCO_2_), respiratory exchange ratio, heart rate and rate of perceived exertion [6, 37, 38, 41]. Li et al. (2024) reported modelling‐based work which evaluated internal loading measures, including forefoot stress, metatarsal stress and hallux stress using finite element simulation.

### 3.5 Commercial Descriptions and Claimed Effects of Rocker Footwear

Commercial footwear brands describe rocker designs in terms of perceived performance benefits, using qualitative language without providing biomechanical metrics or quantitative evidence. In most cases, these descriptions replace explicit geometric characterisation. Claims commonly emphasise enhanced propulsion, efficiency, smoothness of transition and ride quality.

Adidas describes its rocker configurations as enabling ‘smooth and efficient forefoot running,’ ‘smooth roll off’ and ‘forward propulsion.’ ASICS states that its rockered designs ‘propel the runner through the stride’ and create a ‘smooth, efficient ride.’ Brooks' ‘GlideRoll Rocker’ is described as assisting ‘smooth heel to toe transitions’ and reducing ‘underfoot pressure.’ HOKA frequently refers to ‘Smooth MetaRocker’ and ‘Speed‐centric MetaRocker’ concepts, claiming they facilitate ‘aggressive toe off,’ ‘pace pushing movement’ and ‘natural gait transitions’.

On running describes its ‘unique rocker shape’ or ‘agile forefoot rocker’ as promoting ‘propulsive toe off’ and enabling a ‘fast, propulsive ride.’ New Balance refers more generally to a ‘rocker profile’ that provides ‘smoother, natural feeling transitions.’ Saucony mentions a ‘gradual rocker sensation’ but does not specify associated functional claims.

Across all brands, rocker related claims are framed in terms of intended user experience, including propulsion, efficiency, comfort, transition smoothness and running economy, rather than measurable geometric or biomechanical characteristics. In the reviewed materials, no brand provided apex location, rocker angle, curvature radius or other quantifiable operational definitions.

## 4 Discussion

The findings of this review revealed that terminology and descriptions related to rockered footwear varied considerably across academic literature and commercial sources. It also highlighted a range of biomechanical and metabolic variables measured when assessing rockered footwear in runners.

The variation in terminology and descriptions may be due to the absence of a clear classification or threshold specifying when a shoe should be classified as incorporating a ‘rocker’. The absence of a standardised definition for ‘rockers’ undermines the ability to develop evidence‐based recommendations. Hutchins et al. (2009) previously raised concerns that rocker sole may be a misleading term as it theoretically should be limited to describing an addition to the sole or forepart of the shoe. They suggested that rocker profile is a more appropriate generic term, provided that the apex position and profile shape are clearly quantified and many studies do not adequately report specifications [8]. Although the current review found academic sources do describe rockers, none established a measurable threshold and terminology in commercial materials is even more ambiguous. The lack of consistent terminology and reporting across studies makes it difficult to compare findings between investigations and across specific footwear models. Although the absence of a consensus definition for ‘rocker’ likely contributes to this variability, it does not fully account for it as reporting practices remain inconsistent even when geometric features are described.

In other fields, research shows that inconsistent terminology produces ‘blurry’ concepts, which in turn reduces measurement precision and limits practical utility [42, 43]. When a single term is used to describe multiple distinct constructs, findings cannot be reliably compared [44] nor can commercial claims be meaningfully evaluated against academic research. For example, Staunton et al. highlight that ‘external load’ has been used to describe both running distance (volume) and frequency of high‐speed efforts (intensity), illustrating how a single term may represent multiple distinct constructs. This conceptual ambiguity increases the likelihood of generating ‘bad data’, reducing the validity of measurements and product comparisons [45]. As a result, retailers and consumers struggle to differentiate between models because vague descriptors obscure the specific mechanical properties being marketed. Ultimately, if the defining attributes of a rocker cannot be clearly articulated, they cannot be accurately measured, monitored or recommended, fundamentally compromising both clinical decision‐making and in‐store selection. This issue is further compounded by the discrepancy between academic and commercial descriptions of rocker footwear. Although quantitative parameters are commonly reported in research, commercial communications rely predominantly on qualitative language, which may be appropriate for conveying user experience but limits the ability to evaluate or compare these claims against scientific evidence. This disconnect may create challenges for clinicians and researchers when translating evidence into practice and for consumers when interpreting the functional characteristics of footwear.

Beyond definitional issues, it is important to consider how rockers are being used and discussed in practice. A recent survey of clinicians, footwear retailers and coaches reported that most respondents (72%) routinely discuss rockers in their advice [5], although it is not clear what specific objectives these recommendations target. The suggested benefits of rockers promoted by footwear brands predominantly relates to comfort, performance and injury prevention or load management. Comfort has been conceptually prioritised in running footwear research [46] and is commonly promoted as a crucial factor in footwear selection [3]. Limited evidence has focused on comfort of rockered footwear in running with Sobhani et al. [2014] presenting the only available data. Their analysis compared ‘standard’ shoes to footwear modified with a rocker and found runners reported a significant reduction in comfort when using the rockered footwear [37].

The notion that footwear can enhance running performance is well established, with advanced footwear technology (often referred to as ‘super shoes’) shown to improve running performance by approximately 2% [47]. Rather than any single design element, these benefits are widely attributed to a combination of increased longitudinal bending stiffness, advanced midsole foams and to a lesser extent midsole geometries. In contrast, the isolated influence of rocker characteristics on running performance has received comparatively little attention. Only two studies have reported physiological responses to rockered footwear these being Tankink et al. (2024) and Sobhani et al. (2014), both of which added rockers extrinsically to footwear. Notably, neither study demonstrated improvements in running economy, and Sobhani et al. (2014) reported increased oxygen consumption when running in footwear with an added rocker. However, it is important to recognise that these findings may not translate directly to commercial footwear as extrinsic modifications can differ substantially from integrated rocker designs, a distinction made more difficult to evaluate due to the ambiguous and inconsistent terminology identified in this review.

The relationship between rockered footwear and injury risk is perhaps the most nuanced and least straightforward of these claims. Importantly, this review found no evidence reporting prospectively recorded running injuries, nor evidence that could directly determine how rockered footwear influences injury risk in runners. This represents a key gap in the current literature. However, it has been suggested that footwear characteristics can influence loading patterns and either increase or decrease injury risk accordingly, although this effect differs for each runner [48]. This raises the possibility that, for some runners, rockered footwear could confer a reduction in injury risk. However, such interpretations remain speculative in the absence of evidence guiding how running footwear should be appropriately ‘prescribed’ on an individual basis. Current evidence has not conclusively demonstrated that any single footwear characteristic can reduce an individual's injury risk [49]. Although several studies included in this review examined biomechanical outcomes, most commonly kinematic and kinetic measures, these variables have not been shown to reliably predict injury risk in runners [49]. Furthermore, the studies included in this review primarily reported group‐level (mean) data, limiting the ability to identify or interpret individual variability in biomechanical responses. This lack of prospective injury data and limited understanding of individual responses represent important priorities for future research.

To enhance the translation of research into meaningful guidance for runners and practitioners, future work should address several critical areas highlighted by this review. Chief among these challenges is the lack of a clear definition or threshold for what constitutes a ‘rocker’ as well as how this feature should be measured in retail or clinical settings. This ambiguity likely contributes to inconsistency in how rockers are described, evaluated and recommended. Establishing a consensus definition will require structured approaches, such as Delphi methodologies, involving collaboration among researchers, footwear designers, clinicians and retailers. Once developed, this definition should be embedded within the information shared across these groups, such as in product catalogues and technical materials produced by commercial brands for retail partners. Although the use of proprietary brand terminology is inevitable, such terminology should be mapped against a standardised, agreed‐upon lexicon to improve transparency and support clearer communication across academic, commercial and clinical settings. Beyond definitional clarity, future research should prioritise several areas. These include improving the consistency and detail of reporting of rocker geometry and associated biomechanical variables, prospectively examining the influence of rockered footwear on injury risk and evaluating biomechanical responses on an individual basis. To improve comparability across studies, future research should aim to consistently report key geometric parameters (e.g., apex position, rocker angle or curvature radius and toe spring), alongside relevant footwear characteristics such as mass, stiffness and material properties. Because many commercially promoted benefits do not currently align well with published academic outcomes, research may need to prioritise the development and validation of new ways to assess qualitative aspects of the running experience. One example is the work by Lam et al. (2018) [50] who proposed a method to quantify ‘ride’ in running footwear, which could be a useful framework for evaluating rockered designs.

This study presents several strengths and limitations that should be considered when interpreting its findings. With the aim of supporting the translation of research into practice and improving real world footwear decision‐making for runners, it was important to examine the information directly available to consumers; accordingly, a major strength of this study was the inclusion of commercial data alongside academic literature, providing insight into how rocker features are communicated across these domains. However, the footwear market is highly diverse and ever expanding, and because it was not feasible to undertake an exhaustive commercial search, ten brands were selected based on their popularity among participants in the Auckland Marathon. Although this approach offered a pragmatic and contextually relevant sample, it also means that the findings reflect footwear worn in a marathon setting in New Zealand and may not fully represent footwear used in general training or in other geographic contexts. The search strategy was deliberately restricted to the term ‘rocker’, which may have reduced sensitivity by excluding studies and commercial products that describe comparable geometric features using alternative terminology. This is particularly relevant in the commercial domain, where proprietary naming conventions are commonly used and may refer to designs that function similarly to rocker geometries without explicitly using the term ‘rocker’. As a result, some relevant footwear technologies may not have been captured, and the findings should be interpreted as reflecting how the term ‘rocker’ is explicitly used rather than representing all functionally similar design features. This may also have contributed to the absence of certain major brands within the commercial synthesis, where rocker‐like features are present but described using alternative terminology. In addition, as a scoping review, this study is subject to inherent methodological constraints, including the absence of formal quality appraisal; given the objective of mapping and describing what has been assessed in relation to rockered footwear, the review did not synthesise effect estimates or exclude lower‐quality evidence and readers should interpret the reported outcomes with an understanding that the breadth of evidence was charted without evaluation of methodological rigour. The included studies were heterogeneous in terms of study design, footwear interventions and outcome measures, which limited the ability to draw direct comparisons across studies.

## 5 Conclusion

This review identified that rockered footwear is defined in academic literature using a range of explicit geometric measures, yet no consistent or standardised definition was evident. In contrast, commercial descriptions rely largely on qualitative performance‐focused terminology with limited reference to measurable design features. These discrepancies limit the translation of research into practice and hinder evidence‐based clinical recommendations. Anticipated benefits of rockered footwear are difficult to align with, or substantiate through, academic evidence when terminology is inconsistent and definitions remain unclear. Establishing a standardised measurable definition of rocker geometry would enhance comparability across studies and strengthen clinical and commercial application. Future work should prioritise consensus development and evaluate individual responses to rigorously defined rocker designs.

## Author Contributions

**Priyanka Khusal:** conceptualisation, formal analysis, investigation, writing – original draft, writing – review and editing. **Mike Frecklington:** conceptualisation, methodology, writing – review and editing. **Sarah Gardner:** conceptualisation, methodology, writing – review and editing. **Aaron Jackson:** conceptualisation, formal analysis, investigation, methodology, supervision, writing – original draft, writing – review and editing.

## Funding

This study was funded by Auckland University of Technology and ASICS New Zealand for a Summer Student Scholarships awarded to PK. These organisations had no role in the study design, collection, analysis, interpretation of the data or in the decision to submit the article for publication.

## Ethics Statement

The authors have nothing to report.

## Consent

The authors have nothing to report.

## Conflicts of Interest

The authors declare no conflicts of interest.

## Supporting information


Supporting Information S1

Supporting Information S2

## Data Availability

No new data were generated or analysed for this study. This article is a scoping review based on previously published literature.
