# Supplementary material for: Rocker Geometry in Running Footwear: A Scoping Review of Definitions, Commercial Descriptors and Outcome Measures
Source: J Foot Ankle Res. 2026 Aug 19;19(3):e70200. doi: 10.1002/jfa2.70200 (PMC13490342; doi:10.1002/jfa2.70200)
Supplement: Supplementary file 1 [file JFA2-19-e70200-s001.docx]

| **Author (Year)** | **Investigated Function** | **Rocker effects on function** |
| --- | --- | --- |
| Arazpour et al. (2025) | Effects of rocker sole shoes on lower-limb biomechanics during walking and running, including joint kinematics, joint moments and power, muscle activation, plantar pressure redistribution, and spatiotemporal gait parameters to inform clinical prescription and customization | Rocker soles mechanically substitute for distal joint motion, primarily reducing ankle range of motion (ROM) while preserving proximal kinematics. The most consistent effect is a significant reduction in sagittal-plane ankle ROM, largely driven by decreased peak dorsiflexion—by approximately 20% in individuals with Achilles tendinopathy. Heel-to-toe designs further reduce plantarflexion during initial loading (0–10% of gait cycle) and limit ROM in mid-stance, while also decreasing first metatarsophalangeal (MTP) dorsiflexion during push-off. Kinetically, repositioning the rocker apex reduces the external dorsiflexion moment arm, lowering plantarflexion moments, peak ankle power generation, and total ankle work. However, this ankle offloading may shift demand proximally, with evidence of increased peak knee extension moments and impulse during running. Neuromuscular adaptations include delayed gastrocnemius activation, increased tibialis anterior activity (20–35%), and greater gluteus medius activation with smaller rocker radii. Plantar pressure is redistributed away from the metatarsal heads (apex 50–60% shoe length), often increasing heel pressures (up to 47%). Spatiotemporally, reduced push-off power is compensated by shorter stride length, higher cadence, and a shorter stance phase. |
| Boyer et al. (2009) | Mechanisms of adaptation to rockered sole shoes during running, specifically changes in lower-limb kinematics and kinetics at the ankle, knee, and hip joints, including joint angles, ground reaction forces, joint moments, joint power, stance time, and running speed | Rocker-soled footwear alters lower-limb biomechanics primarily through distal adaptations at the ankle while preserving proximal joint patterns. The sagittal curvature induces greater ankle dorsiflexion at heel-strike and mid-stance and reduced plantarflexion at toe-off, whereas knee and hip kinematics remain largely unchanged, suggesting compensatory ankle adjustments to maintain preferred proximal movement patterns. Kinetically, the rocker design reduces mechanical demand at the ankle, with significant decreases in external dorsiflexion, plantarflexion, and inversion moments (dorsiflexion moments reduced by ~12%), as well as lower peak negative and positive ankle joint powers. Knee and hip joint moments remain unchanged, although peak positive knee power increases slightly as compensation, resulting in an overall reduction in total positive joint power across the lower limb. Ground reaction forces shift, with lower first medial and anterior (push-off) peaks, greater posterior force during early stance (first 20%), and no differences in vertical impact peaks or loading rates. Spatiotemporally, runners adopt a slightly slower self-selected speed (2.5 vs. 2.6 m/s) and correspondingly longer stance time (293 vs. 285 ms). |
| Chen et al. (2022) | Effects of rocker-soled shoes on lower-extremity biomechanics during functional tasks, including hip, knee, and ankle joint kinematics across sagittal, frontal, and transverse planes, and muscle activation patterns (vastus medialis, vastus lateralis, biceps femoris, gastrocnemius) during walking, running, stair negotiation, cutting, and jumping | Rocker-soled shoes act primarily through induced instability and altered roll-over mechanics. Their curved anterior–posterior profile creates a relatively unstable base of support, often prompting increased external ankle rotation to enhance medial–lateral stability. The rocker shape facilitates earlier roll-off during stance, assisting forward progression and reducing effort in tasks such as stair ascent, while the smaller apex contact area—particularly during descent—increases neuromuscular demand for stabilization. Kinematically, these shoes reduce ankle dorsiflexion during stair ascent and jumping ascent, increase plantarflexion during stair ascent, and increase transverse-plane external rotation across walking, running, and stair negotiation. At the hip, peak extension and external rotation during walking are reduced, while knee joint angles generally show minimal change. Muscle activation patterns shift accordingly: vastus medialis and lateralis activity increases to stabilize the knee, biceps femoris activation decreases alongside reduced hip extension, and medial gastrocnemius activation decreases during ascent tasks due to mechanical assistance from the rocker. During cutting and landing tasks, instability further elevates quadriceps demand and may aid impact absorption. |
| Frigg et al. (2016) | Effects of rocker bottom shoes on walking biomechanics across clinical and healthy populations, assessed using the Relative Midfoot Index (RMI), forefoot maximal force, and walking speed to evaluate rollover function, forefoot loading, and gait performance following ankle and hindfoot surgery | The Relative Midfoot Index (RMI), forefoot maximal force, and walking speed are key measures used to evaluate the biomechanical effects of rocker shoes. The RMI, which quantifies the depth of the midfoot “valley” relative to hindfoot and forefoot forces, typically approaches one in normal triphasic gait but decreases toward zero in pathological gait. Rocker shoes significantly lowered the RMI in all participants, even causing healthy individuals’ values to converge with those of patients at approximately 0.4–0.5, suggesting a paradoxical shift toward a less optimal rollover pattern. Forefoot maximal force increased when wearing any shoes compared to barefoot walking, but there was no significant difference between standard running shoes and rocker bottom shoes, indicating that the rocker design did not further reduce forefoot loading. Similarly, walking speed improved modestly with footwear but did not differ between shoe types. Overall, while footwear benefits gait performance and load distribution, rocker bottom shoes offered little additional advantage over high-quality running shoes, even for individuals with ankle fusions. |
| Freitag et al. (2023) | Effects of heel and forefoot rocker shoe curvature on pelvic abductor muscle activity (gluteus medius and gluteus maximus) during running under different conditions (flat laboratory surface, treadmill running at 0%, 24% uphill, and 24% downhill gradients) | Rocker shoes influence muscle activation primarily by manipulating the radius of curvature at the forefoot, with a smaller radius creating a reduced support surface that increases joint instability. This instability compels the neuromuscular system to compensate, particularly by increasing activation of the pelvic abductors, including the gluteus medius (GMed) and gluteus maximus (GMax). Both the amplitude and duration of muscle activation rise as the rocker becomes more pronounced, with GMed consistently showing higher peak and average activity than GMax to support coronal plane pelvic alignment during running. Environmental factors further modulate these effects: running overground on a hard, flat surface induces greater activation than treadmill running, uphill running produces the weakest and shortest activation due to lower velocity, and downhill running does not increase activation compared to flat overground conditions. Functionally, these compensatory activations stabilize the pelvis during single-limb support and may help prevent injuries such as iliotibial band syndrome by improving hip control and reducing excessive adduction and internal rotation. |
| Han et al. (2025) | Effects of rocker bottom shoes on ankle and hindfoot biomechanics during walking, including talocrural and subtalar joint dorsiflexion–plantarflexion range of motion, peak joint angles, and dynamic hindfoot posture measured using biplanar fluoroscopy | Rocker bottom shoes (RBS) influence ankle and hindfoot biomechanics by substituting natural ankle motion with their thick, sagittally curved sole, which induces a rolling motion and reduces the motion demands on the ankle joint. This results in a significant decrease in ankle dorsiflexion–plantarflexion range of motion, from 17.9° ± 6.5° barefoot to 10.6° ± 5.2° in RBS, and a similar reduction in total hindfoot motion from 15.5° ± 3.0° to 8.7° ± 3.3°. Peak dorsiflexion angles are limited, with the ankle and hindfoot adopting slightly plantarflexed postures during late stance, and the subtalar joint also becoming more plantarflexed (−7.4° vs −4.0° barefoot). By restricting ankle motion, the RBS redistributes sagittal-plane rotation to adjacent joints, with trends toward increased subtalar motion (11.3° vs 8.5° barefoot) compensating for the reduced ankle excursion. Clinically, these effects may benefit individuals with anterior ankle impingement or arthritis by limiting joint excursion, though increased subtalar motion could exacerbate pre-existing subtalar pathology. |
| Li et al. (2017) | Effects of toe spring magnitude and forefoot landing angle on metatarsal and hallux stress distribution during running, with a focus on identifying configurations that minimise injury-related stress in the forefoot | The toe spring of a rocker shoe facilitates a rolling motion that promotes rapid forefoot liftoff and reduces sustained loading on the metatarsals, helping to minimize cumulative microdamage and the risk of stress fractures. Higher toe spring designs (8 cm) decrease peak stress and stress concentration in the metatarsals compared to lower toe spring shoes (6.5 cm), particularly reducing peak stress in the second metatarsal as landing angles increase. The curved design also accommodates natural metatarsophalangeal joint motion and protects the hallux by lowering pressure within the toe box, preventing repeated impacts that can cause subungual hematomas. Effectiveness depends on landing angle: minimum stress for the first and third metatarsals occurs at approximately 10°, while angles ≥15° increase stress on the hallux and lateral metatarsals due to bending and shear strain. Optimal configurations to reduce forefoot injury include a higher toe spring (8 cm), a landing angle near 10°, and avoiding angles ≥15°. |
| Lin et al. (2024) | Plantar force (% body weight), difference in plantar force (DPF), temporal gait events (T1, T2), stance phase duration (DSP), overall duration (OD), ankle dorsiflexion angle, foot-to-floor angle; therapeutic and preventative function to reduce plantar loading and stress concentration during walking and jogging | Rocker shoes significantly alter plantar forces, spatiotemporal parameters, and ankle-foot kinematics to facilitate smoother roll-over and reduce stress concentrations. During walking, plantar force (PF) is reduced by 16–32% of body weight compared to barefoot, whereas jogging often produces higher PF due to faster force transmission and reduced shock absorption. The difference in plantar force (DPF) between the foot-shoe interface and vertical ground reaction force is also lower in rocker shoes, whereas flat shoes can exhibit PF greater than VGRF. Temporal adaptations include shorter heel-contact-to-P1 durations (T1) during walking, slightly longer T2 durations during jogging, and an overall lengthened stance phase, particularly with softer rocker materials. Ankle dorsiflexion and foot-to-floor angles at heel contact are increased due to sole thickness, inducing earlier ankle rotation. Therapeutically, rocker shoes convert vertical kinetic energy into rotational motion, utilize rear and front curve rolling to minimize joint bending, redistribute weight via the curved sole to expand contact area, and optimize stiffness—softer materials enhance walking shock absorption, while denser materials better handle jogging forces. |
| Mei et al. (2019) | Gait kinematics, plantar pressure distribution, stance time, running speed, and forefoot/toe loading, with emphasis on stimulating toe-gripping function and altering forefoot biomechanics during running | The regionalised rocker functions as an unstable stimulus designed to actively engage the toe-gripping mechanism, particularly at the hallux, by positioning the rocker vertex beneath its central pressing point. This placement compels active push-off during toe-off to maintain stability, effectively expanding the forefoot load-bearing area. In the soft rocker configuration (SS), this results in significantly increased maximal force and peak pressure at the big toe, accompanied by a marked reduction in maximal force, peak pressure, and force-time integral across the medial, central, and lateral metatarsal regions, thereby redistributing plantar load away from the forefoot. The soft material further enhances total support area. Functionally, SS produces a significantly higher running speed and shorter stance time, likely due to facilitated push-off and a quicker gait transition. Kinematically, SS increases peak ankle plantarflexion and sagittal range of motion via windlass activation, while largely preserving overall joint motion aside from reduced hip abduction and increased hip rotation ROM. In contrast, the hard rocker (SH) induces laterally shifted plantar loading and greater compensatory strategies, including increased hip flexion, hip external rotation, and altered knee and ankle mechanics, highlighting material stiffness as a critical determinant of biomechanical response. |
| Munim et al (2019) | Effects on lower-limb biomechanics during walking and running: joint angles, moments and power; plantar pressure; muscle activity; spatiotemporal parameters; oxygen consumption; perceived comfort | Rocker shoes act as a mechanical substitute for natural joint motion, primarily at the ankle, by reducing sagittal-plane ankle range of motion and peak dorsiflexion while increasing dorsiflexion at heel strike, which facilitates earlier forward roll. Peak plantarflexor moments and ankle joint power are decreased, often prompting compensatory increases in knee work to maintain forward progression. The curved sole redistributes plantar pressure, lowering peak loads at the forefoot and toes while shifting forces to the midfoot and rearfoot. Muscle activation adapts to the unstable surface, with increased activity in the tibialis anterior, gluteus medius, gluteus maximus, and quadriceps to preserve postural control and manage altered striking patterns, though smoother roll-off may reduce demand on some muscles. Spatiotemporal adjustments include shorter stride length, increased cadence, decreased stance time, and possible swing-time increases. Energy cost is minimally affected during walking but may rise during running due to shoe mass, while perceived comfort can decline because of instability and changes in foot-strike patterns. |
| Nakagawa et al (2018) | To investigate whether wearing unstable rocker (MBT) shoes enhances recovery from marathon-induced muscle damage by accelerating restoration of muscle hardness, strength, and perceived fatigue | Rocker shoes, such as MBT footwear, appear to enhance post-exercise recovery by accelerating the restoration of muscle mechanical properties, strength, and perceived fatigue. The instability of the rocker sole increases lower-limb muscle activity and ankle range of motion during walking, stimulating the venous muscle pump and enhancing blood flow, which facilitates faster clearance of metabolic byproducts and accelerates recovery of muscle hardness (strain ratio) in the medial gastrocnemius, vastus lateralis, tibialis anterior, and biceps femoris. Recovery of muscle strength, particularly knee flexion torque, is also improved, with strength returning to baseline by the first day post-race in the MBT group, whereas controls had not recovered by day eight. This may be partly due to reduced knee flexion moments and range of motion during walking, decreasing load on the knee flexors and allowing faster restoration. Correspondingly, subjective fatigue scores improved more rapidly in the MBT group, with significantly lower fatigue reported by days seven and eight, likely reflecting the accelerated physiological recovery processes. |
| Sanchez-Gomez et al. (2020) | To compare the effects of NIRO versus TMEO inverted rocker orthoses on electromyographic (EMG) activity of the gastrocnemius medialis and lateralis during running | Rocker orthoses influence running biomechanics by mechanically limiting first metatarsophalangeal joint (IMTPJ) mobility, disrupting natural energy-storage mechanisms and increasing muscular demand. The toe-metatarsal extension orthosis (TMEO), a flat extension under the first ray, restricts IMTPJ dorsiflexion and consequently impairs the windlass mechanism, reducing the medial longitudinal arch’s ability to store and release elastic energy during heel-off. As a result, the gastrocnemius medialis and lateralis must increase EMG activity to compensate for the loss of elastic recoil and stabilize propulsion. Similarly, the non-inverted rocker orthosis (NIRO), designed with smooth inverted rocker edges under the IMTPJ, introduces joint instability that is transferred to the triceps surae, producing even greater increases in gastrocnemius activation—particularly with the 8 mm thickness. Both orthoses significantly elevate EMG activity compared to shoes alone, and thicker designs amplify this response. This increased muscular cost may reduce running economy by elevating neuromuscular demand and oxygen consumption at a given speed. |
| Sobhani et al. (2013) | Ankle joint biomechanics during slow running and walking: primary outcome was peak ankle plantarflexion moment during terminal stance. Secondary outcomes included ankle plantarflexion impulse, ankle power, ankle angles, knee and hip joint moments, and EMG (timing and amplitude) of triceps surae and tibialis anterior | Rocker shoes reduce ankle loading by altering the leverage of ground reaction forces around the joint. Positioning the rocker apex proximal to the metatarsophalangeal joint decreases the external dorsiflexion moment arm, thereby reducing the external dorsiflexion moment that must be countered during late stance. This results in a significantly lower internal plantarflexion moment generated by the triceps surae and a corresponding reduction in plantarflexion moment impulse during both walking and running. The rocker profile also acts as a mechanical substitute for ankle excursion, promoting earlier roll-off, reducing peak dorsiflexion angles, and significantly decreasing peak ankle power generation during propulsion. Neuromuscularly, while peak triceps surae amplitude is not systematically altered, the timing of peak activation is delayed by approximately 2% during walking, likely due to facilitated forward roll. In contrast, tibialis anterior activity increases significantly (≈20% or 64.7 mV). These adaptations appear localized to the ankle, with no significant changes observed in peak knee or hip joint moments during walking or running. |
| Sobhani et al. (2014) | Plantar pressure distribution during running, including peak pressure (PP), maximum mean pressure (MMP), and force–time integral (FTI) across foot regions, as well as perceived shoe comfort and exploratory analysis of strike pattern effects | Rocker profiles substantially redistribute plantar pressure during running, producing clinically relevant forefoot offloading alongside increased heel loading. Peak pressure (PP), maximum mean pressure (MMP), and force-time integral (FTI) are significantly reduced in the central and lateral forefoot (17–28%), with medial forefoot MMP and FTI decreasing by approximately 11% and 12%, respectively—effects beneficial for conditions such as metatarsalgia or stress fractures. However, this redistribution results in marked increases in heel loading, with PP, MMP, and FTI rising by 47%, 22%, and 52%, likely due to the stiffened sole providing less cushioning at initial contact. While first toe pressures remain unchanged, PP in the lesser toes increases by 10%, possibly compensating for reduced metatarsophalangeal propulsion. Despite these biomechanical benefits, rocker shoes are perceived as significantly less comfortable (VAS 33.3 vs. 76.1), partly due to increased mass (~317 g heavier per pair) and limited familiarization time. Importantly, forefoot offloading and heel loading occur regardless of strike pattern, although forefoot/midfoot strikers may shift toward a rearfoot strike and experience greater heel pressure increases. |
| Sobhani et al. (2014) | Metabolic and physiological responses during running: VO₂, VCO₂, respiratory exchange ratio (RER), heart rate (HR), rate of perceived exertion (RPE) | Running in rocker shoes results in a significant increase in oxygen consumption (VO₂) compared to both standard and minimalist footwear, with values averaging 4.5% higher than standard shoes and 5.6% higher than minimalist shoes. This increase is largely attributed to the greater mass of the rocker shoes, which averaged 858 g per pair compared to 541 g for standard shoes, accounting for approximately 3.1% of the VO₂ increase, while additional factors include runners’ inexperience with the rocker design and biomechanical changes in ankle movement or spatiotemporal gait parameters. Despite these increases in energy expenditure, the respiratory exchange ratio (RER) remains unchanged at a mean of 0.96, confirming that participants maintained sub-maximal intensity. Heart rate is also unaffected, with a mean of 151.97 bpm similar across shoe conditions, and the rate of perceived exertion (RPE) remains at a “light” intensity (mean 11.31 on the Borg scale), indicating that runners do not perceive a substantial increase in effort despite the higher physiological cost imposed by the rocker shoe. |
| Sobhani et al. (2015) | Gait kinematics, kinetics, and spatiotemporal outcomes (ankle plantarflexion moment, knee flexion moment, hip flexion moment, ankle power, ankle dorsiflexion), EMG (gastrocnemius, soleus, tibialis anterior), speed, step length, cadence, stance time, and pain | Rocker shoes significantly alter lower-limb biomechanics during walking and running, primarily by reducing ankle joint loading. The reduced external dorsiflexion moment leads to a significant decrease in internal ankle plantarflexion moment (PFM) of 13% in both running and walking, with corresponding reductions in PFM impulse of 15% during running and 19% during walking. Peak ankle power generation during propulsion is also significantly reduced (23% in running and 21% in walking). While peak knee flexion moments remain unchanged in both activities, hip flexion moment is reduced by 8% during walking but unaffected during running. Kinematically, the rocker design promotes earlier roll-off, resulting in reduced peak ankle dorsiflexion (11% in running and 20% in walking) and a 14% reduction in total ankle range of motion during walking, with no change observed during running. Electromyography findings show no significant changes in peak triceps surae activity in either condition, although the timing of peak lateral gastrocnemius activity is delayed by approximately 4% of the gait cycle during running. Tibialis anterior peak activity increases by 35% during walking but does not change during running. Spatiotemporal parameters—including speed, step length, and cadence—remain unchanged, as participants maintained self-selected speeds, although stance time is slightly but significantly reduced in both walking and running. Despite successfully unloading the Achilles tendon, rocker shoes do not produce immediate changes in Achilles tendon pain after 10–15 minutes of wear, suggesting that longer-term use and sustained relative unloading are required to facilitate tissue healing. |
| Sobhani et al. (2017) | Lower-limb biomechanics during running, including joint work distribution (ankle, knee, hip), joint moments, kinematics, stance time, and influence of strike pattern (rearfoot vs midfoot) on rocker shoe effects. | The mechanism underlying the rocker shoe’s effects is primarily related to its specific geometry, particularly a stiffened rocker profile with the apex positioned more proximally at 53% of shoe length compared to 65% in standard shoes. This proximal apex reduces the external dorsiflexion moment arm of the ground reaction force (GRF) at the ankle, shortening the lever arm and consequently decreasing the demand on the triceps surae to generate an internal plantarflexion moment (PFM). At the same time, the posterior shift of the GRF relative to the knee increases the external knee moment arm, requiring greater knee extensor contribution and increasing extension moments and mechanical work at the knee. As a result, joint work is redistributed proximally: during running, positive ankle work decreases by 16% and negative work by 32%, while positive knee work increases by 14% and net knee work by 19%, with a 17% reduction in net hip work. Overall, total lower-limb net work is reduced by 0.146 J/kg compared to standard shoes. Mechanically, peak ankle PFM decreases by 11% and PFM impulse by 12%, whereas the knee shows a 6% increase in peak extension moment and a 12% increase in extension moment impulse; hip flexion moment impulse decreases by 13%. Kinematically, peak ankle dorsiflexion is reduced by 7% as the rocker profile assists forward roll-over, reducing required ankle range of motion, and stance time is shortened by approximately 3% (around 0.006 seconds) during running. The magnitude of these effects is influenced by foot strike pattern: although the direction of change is consistent across runners, reductions in ankle work and PFM are nearly twice as large in midfoot strikers compared to rearfoot strikers. The proximal apex may also destabilise a midfoot landing, encouraging a shift toward rearfoot striking. Given that midfoot strikers typically experience higher Achilles tendon loads, this greater unloading effect may provide enhanced therapeutic benefit for this group. |
| Tankink et al. (2024) | Running biomechanics: positive/negative ankle work, share of joint work (%), ankle angles, angular velocity, plantar flexion moments, power generation, vertical and anterior-posterior GRF, metabolic power, step frequency, stance time | Rocker shoes redistribute mechanical demand across the lower limb by altering ankle joint work and kinetics. Individually optimized rocker profiles—typically featuring a more distal apex position (approximately 75–84% of shoe length)—significantly increase both positive and negative ankle work compared to standard rocker settings, thereby increasing the ankle’s proportional contribution to total lower limb work and shifting load away from the hip and knee. The more distal apex creates a larger external moment arm, delaying foot rotation and producing greater peak dorsiflexion, as well as higher plantarflexion angular velocity during propulsion. The increased ground reaction force (GRF) moment arm requires greater internal plantarflexion moments, resulting in elevated ankle power absorption and generation. Optimized rockers also shift the anterior–posterior point of force application distally during mid-stance (37–59%) and alter vertical GRF patterns in late stance (72–97%). However, despite increasing ankle work, these adaptations do not necessarily improve running economy, as greater triceps surae force production, increased ankle range of motion, and added shoe mass (≈4% higher metabolic cost; >200 g heavier per foot) may offset theoretical elastic energy benefits. Notably, step frequency and stance time remain unchanged, indicating preserved spatiotemporal gait patterns despite altered internal mechanics. |
| Trama et al. (2019) | Running biomechanics: EMG of gastrocnemius and vastus lateralis (pre-activation & stance), ground reaction forces (impact peak, active peak, VLR, impact frequency), soft tissue vibrations (Accpeak, Pmean, Pmax, HFratio, DAMP), effect of running speed | Rocker shoes had minimal effects on muscular activity, ground reaction forces (GRF), and soft tissue vibrations during running. Electromyography of the gastrocnemius medialis (GAS) and vastus lateralis (VL) showed no significant changes in pre-activation or stance-phase activity, likely because the kinematic alterations were too small and the shoes’ thickness and material were identical to standard footwear. Similarly, GRF parameters—including impact peak, active peak, vertical loading rate, and impact frequency—remained unchanged, suggesting that previously reported reductions in other studies were attributable to differences in sole material or thickness rather than rocker geometry. Soft tissue vibrations in GAS and VL, including peak and mean acceleration, maximal power, high-frequency ratio, and damping coefficient, were also unaffected, as neither the input shock (GRF) nor muscle activity changed. Running speed, rather than shoe design, was the dominant factor, increasing muscular activity, GRF peaks, vibration power, and high-frequency vibrations, with the neuromuscular system correspondingly enhancing damping to mitigate these effects. |
